# Cryphonectriaceae associated with rust-infected *Syzygium jambos* in Hawaii

**DOI:** 10.3897/mycokeys.76.58406

**Published:** 2020-12-31

**Authors:** Jolanda Roux, Gilbert Kamgan Nkuekam, Seonju Marincowitz, Nicolaas A. van der Merwe, Janice Uchida, Michael J. Wingfield, ShuaiFei Chen

**Affiliations:** 1 Department of Plant and Soil Sciences, Forestry and Agricultural Biotechnology Institute (FABI), University of Pretoria, Pretoria 0028, South Africa University of Pretoria Pretoria South Africa; 2 Department of Biochemistry, Genetics and Microbiology, FABI, University of Pretoria, Pretoria 0028, South Africa University of Hawaii at Manoa Honolulu United States of America; 3 Department of Plant and Environmental Protection Sciences, Tropical Plant Pathology Program, College of Tropical Agriculture and Human Resources, University of Hawaii at Manoa, Honolulu, Hawaii 96822, USA University of Pretoria Zhanjiang China; 4 China Eucalypt Research Centre (CERC), Chinese Academy of Forestry (CAF), ZhanJiang, 524022, GuangDong Province, China China Eucalypt Research Centre, Chinese Academy of Forestry ZhanJiang China

**Keywords:** *Austropuccinia psidii*, fungi, genetic diversity, Myrtales, pathogen introductions

## Abstract

*Syzygium jambos* (Myrtales, Myrtaceae) trees in Hawaii are severely affected by a rust disease caused by *Austropuccinia psidii* (Pucciniales, Sphaerophragmiaceae), but they are commonly co-infected with species of Cryphonectriaceae (Diaporthales). In this study, *S. jambos* and other trees in the Myrtales were examined on three Hawaiian Islands for the presence of Cryphonectriaceae. Bark samples with fruiting bodies were collected from infected trees and fungi were isolated directly from these structures. Pure cultures were produced and the fungi were identified using DNA sequence data for the internal transcribed spacer (ITS) region, part of the β-tubulin (*BT1*) gene and the transcription elongation factor-1α (*TEF1*) gene. Five species in three genera of Cryphonectriaceae were identified from Myrtaceae tree samples. These included *Chrysoporthe deuterocubensis*, *Microthia havanensis* and three previously-unknown taxa described here as *Celoporthe hauoliensis* sp. nov., *Cel. hawaiiensis* sp. nov. and *Cel. paradisiaca* sp. nov. Representative isolates of *Cel. hauoliensis*, *Cel. hawaiiensis*, *Cel. paradisiaca*, *Chr. deuterocubensis* and *Mic. havanensis* were used in artificial inoculation studies to consider their pathogenicity on *S. jambos*. *Celoporthe hawaiiensis*, *Cel. paradisiaca* and *Chr. deuterocubensis* produced lesions on young *S. jambos* trees in inoculation trials, suggesting that, together with *A. psidii*, they may contribute to the death of trees. Microsatellite markers were subsequently used to consider the diversity of *Chr. deuterocubensis* on the Islands and thus to gain insights into its possible origin in Hawaii. Isolates of this important Myrtaceae and particularly *Eucalyptus* pathogen were found to be clonal. This provides evidence that *Chr. deuterocubensis* was introduced to the Hawaiian Islands as a single introduction, from a currently unknown source.

## Introduction

Fungi in the Cryphonectriaceae (Diaporthales) include at least twenty-three genera of bark-, wood- and leaf-infecting fungi (Gryzenhout et al. 2009, 2010; Begoude et al. 2010; Vermeulen et al. 2011, 2013; Crous et al. 2012; Chen et al. 2013a, b, 2016, 2018; Crane and Burgess 2013; Beier et al. 2015; Ali et al. 2018; Ferreira et al. 2019; Jiang et al. 2020; Wang et al. 2020). They occur on trees and shrubs in various parts of the world and include saprophytes, facultative parasites and important pathogens of woody plants (Gryzenhout et al. 2009). Pathogens in the family reside mainly in the genera *Cryphonectria* and *Chrysoporthe* and include important agents of tree disease, both in natural forest ecosystems, as well as in intensively-managed plantations (Wingfield 2003; Gryzenhout et al. 2009; Wang et al. 2020). These fungi generally have yellow to orange or brown stromata and these structures turn purple in 3% potassium hydroxide (KOH) or yellow in lactic acid (Gryzenhout et al. 2006c, 2009; Jiang et al. 2020).

The Cryphonectriaceae infect trees and shrubs residing in more than 100 species in at least 26 families and 16 orders of plants worldwide (Gryzenhout et al. 2009; Jiang et al. 2020; Wang et al. 2020). The chestnut blight pathogen, *Cryphonectria parasitica* (Murrill) M.E. Barr is the best-known tree-killing pathogen in the family. It is native to Asia and outbreaks of the disease in North America and Europe have caused the virtual extinction of endemic populations of chestnut trees on these two continents (Anagnostakis 1987; Heiniger and Rigling 1994; Gryzenhout et al. 2009). Other important pathogens in the Cryphonectriaceae include: *Chrysoporthe cubensis* (Bruner) Gryzenh. & M.J. Wingf., which is native to South and Central America and causes a canker disease of *Eucalyptus* species in West Africa and South America (Alfenas et al. 1983; Gryzenhout et al. 2004, 2009; Roux and Apetorgbor 2010); *Chrysoporthe deuterocubensis* Gryzenh. & M.J. Wingf., native to Southeast Asia and causal agent of a canker disease of *Eucalyptus* species in Africa, Australia, China and Hawaii (Davison and Coates 1991; Roux et al. 2005; Nakabonge et al. 2006; Zhou et al. 2008; Chen et al. 2010; Van der Merwe et al. 2010; Wang et al. 2020); and *Chrysoporthe austroafricana* Gryzenh. & M.J. Wingf., endemic to Africa and causal agent of a canker disease of *Eucalyptus*, *Syzygium* and *Tibouchina* species in southern and eastern Africa (Wingfield et al. 1989; Myburg et al. 2002a; Gryzenhout et al. 2004; Roux et al. 2005; Nakabonge et al. 2006).

Hawaii, in the central Pacific Ocean, is comprised entirely of islands and is the northernmost island group in Polynesia (Little and Skolmen 1989). The vegetation is multivariate including many forest types that cover more than 41% of the State’s total land area (Anonymous 2003). Hawaii’s forests broadly comprise native forest and plantations of non-native trees, interspersed with stands of non-native, invasive tree species. Native forests are dominated by *Metrosideros polymorpha* Gaudich. (Myrtaceae, Myrtales) and *Acacia koa* A. Gray (Fabaceae, Fabales) trees, whereas plantations of non-native trees include various conifers and many tree species (mostly *Eucalyptus*) that reside in the Myrtaceae (Anonymous 2003). Eight species of indigenous Myrtaceae and more than 200 non-native Myrtaceae have been recorded from the Islands (Loope 2010).

In April 2005, a rust disease caused by *Austropuccinia psidii* G. Winter (syn. *Puccinia psidii*, Sphaerophragmiaceae, Pucciniales), was detected on the Island of O’ahu (Uchida et al. 2006; Loope 2010). The pathogen spread rapidly and, consistent with its broad host range in the Myrtaceae (Coutinho et al. 1998; Glen et al. 2007; Carnegie et al. 2016), has been reported to cause disease on at least five native and fifteen non-native Hawaiian species. Of these, the non-native and invasive *Syzygium jambos* (rose apple) has been especially severely affected by the disease (Loope 2010). Instances of crown death of *S. jambos* are common and, in some cases, large areas of trees have died (Loope 2010).

During a casual inspection of rust-infected *S. jambos* in Hawaii by M.J. Wingfield during August 2011 (unpublished data), fruiting bodies of fungi resembling species in the Cryphonectriaceae were observed on the stems and branches of dying trees. This raised interest as very little was known regarding the diversity and distribution of the Cryphonectriaceae infecting Myrtaceae on the Hawaiian Islands. Two species are known to occur and these include, *Chr. deuterocubensis*, collected from cankers on *Eucalyptus* species on the Islands of Kauai and Hawaii (Gryzenhout et al. 2006a, 2009; Van der Merwe et al. 2010) and *Microthia havanensis* (Bruner) Gryzenh. & M.J. Wingf., first found on *Eucalyptus* species grown on the same Islands (Gryzenhout et al. 2006a).

The dramatic death of *S. jambos* in Hawaii could be caused solely by *A. psidii*, but the extent of the rapid die-back of branches and stems raised the question as to whether other pathogens, such as the Cryphonectriaceae, might be involved. The aim of this study was, thus, to identify species of Cryphonectriaceae on rust-infected *S. jambos*, as well as on some other species of Myrtaceae. Furthermore, pathogenicity tests were used to consider the possibility that species in the Cryphonectriaceae might contribute to the death of trees that had become infected and were consequently stressed by *A. psidii*. The genetic diversity of a collection of the most commonly isolated Cryphonectriaceae species was also characterised to gain insight into its possible origin in Hawaii.

## Materials and methods

### Collection of samples and fungal isolation

Surveys for Cryphonectriaceae were conducted in Hawaii during July 2012. Samples were collected mainly from non-native *S. jambos* trees infected by *A. psidii*, but also from various native and non-native Myrtaceae, on the Islands of Maui, O’ahu and Hawaii. Samples were collected using an unstructured approach where the areas sampled were determined by the time available for collections to be made on the three selected Islands. On each of the Islands, two to three sites, where rust-infected trees had previously been found, were selected and surveyed during the course of a single day. As much as possible of each Island was also covered by driving along main roads and sampling at regular intervals where *S. jambos* plants were observed.

The presence on samples of fruiting structures (ascostromata, conidiomata), typical of the Cryphonectriaceae, was ascertained using a 10× magnification hand lens. Pieces of bark bearing these fruiting structures were excised from infected stems and branches and placed in separate brown paper bags for each tree sampled. Samples from each Island were labelled and placed in plastic bags to prevent desiccation and to promote sporulation of the fungi. Isolations and purification of the Cryphonectriaceae from the wood samples followed the technique described by Chen et al. (2011). All isolates used in this study were deposited in the culture collection (CMW) of the Forestry and Agricultural Biotechnology Institute (FABI, www.fabinet.up.ac.za), University of Pretoria, Pretoria, South Africa. Representative isolates, including ex-type cultures, were deposited in the culture collection (CBS) of the Westerdijk Fungal Biodiversity Institute, Utrecht, The Netherlands. Dried specimens of cultures were deposited in the National Collection of Fungi (PREM), Roodeplaat, Pretoria, South Africa.

### DNA extraction, PCR amplification and sequencing

DNA was extracted from all isolates using PrepMan Ultra Sample Preparation Reagent kits (Applied Biosystems, California, USA) following the manufacturer’s instructions. An Eppendorf Mastercycler (Merck, Germany) was used for PCR amplification of the nuclear rDNA region encompassing the internal transcribed spacer regions (ITS1, ITS2) and 5.8S gene of the ribosomal RNA (ITS) operon, part of the β-tubulin gene (*BT1*) and the transcription elongation factor-1α gene (*TEF1*). The ITS was amplified using primers ITS1 and ITS4 (White et al. 1990), the *BT1* using primers βt1a and βt1b (Glass and Donaldson 1995) and *TEF1* using primers EF728F and EF986R (Carbone and Kohn 1999). The PCR reaction mixtures and thermal cycling conditions were the same as described previously for the ITS, *BT1* (Chen et al. 2011, 2013a) and *TEF1* gene regions (Vermeulen et al. 2013).

A 5 µl aliquot of the PCR products was pre-stained with GelRed^TM^ Nucleic Acid Gel stain (Biotium, Hayward, USA), separated on 1% agarose gels and visualised under UV light. PCR products were purified using Sephadex G-50 Gel (Sigma-Aldrich), following the manufacturer’s instructions. The concentrations of the purified PCR products were determined using a Nanodrop ND-1000 Spectrophotometer (Nanodrop Technologies, Rockland, USA). Sequencing reactions were performed using the Big Dye cycle sequencing kit with Amplitaq DNA polymerase FS (Perkin-Elmer, Warrington, UK), following the manufacturer’s protocols, on an ABI PRISM 3100 Genetic Analyzer (Applied Biosystems). Protocols for sequencing PCR amplicons were the same as those described by Chen et al. (2011) and both DNA strands were sequenced for each gene region. Sequences of both DNA strands for each isolate were examined visually and combined using the programme Sequence Navigator v. 1.01 (ABI PRISM, Perkin Elmer). The ITS and *BT1* gene regions were sequenced for all isolates used in this study. The *TEF1* gene region was sequenced for selected isolates in genera for which this region was required for species-level identification.

### Phylogenetic analyses

A preliminary identification of the isolates was obtained by performing a similarity search (standard nucleotide BLAST) against the GenBank database (http://www.ncbi.nlm.nih.gov) using the ITS and *BT1* sequences. The BLAST results showed that the isolates obtained in the current study grouped in the genera *Celoporthe*, *Chrysoporthe* and *Microthia*.

For analyses of the ITS and *BT1* sequences of isolates from Hawaii, the datasets of Wang et al. (2020) were used as templates. Sequences of the ITS and *BT1* gene regions were analysed separately and in combination. A partition homogeneity test (PHT), as implemented in PAUP (Phylogenetic Analysis Using Parsimony) v. 4.0b10 (Swofford 2003), was used to determine whether there was conflict between the datasets, prior to performing combined analyses in PAUP (Farris et al. 1995; Huelsenbeck et al. 1996). Two isolates of *Diaporthe ambigua* (CMW5288 and CMW5587), residing in the Diaporthaceae (Diaporthales), were used as outgroups.

For isolates that grouped in *Celoporthe*, based on ITS and *BT1* gene sequences, *TEF1* sequences were required to obtain accurate species-level identifications (Chen et al. 2011; Vermeulen et al. 2013). The ITS, *BT1* and *TEF1* gene regions were analysed separately and in combination. This made it possible to determine the phylogenetic relationships amongst the isolates from Hawaii and all 10 previously described *Celoporthe* species (Nakabonge et al. 2006; Chen et al. 2011; Vermeulen et al. 2013; Ali et al. 2018; Wang et al. 2018). A PHT was used to determine if conflict existed amongst the ITS, *BT1* and *TEF1* datasets (Farris et al. 1995; Huelsenbeck et al. 1996). Two isolates of *Holocryphia capensis* (CMW37329 and CMW37887) were used as outgroups.

The sequences for each of the single gene datasets, as well as for a combined dataset consisting of two or three gene regions, were aligned using MAFFT online v. 7 (http://mafft.cbrc.jp/alignment/server/) (Katoh and Standley 2013) and applying the iterative refinement method (FFT-NS-i setting). The alignments were edited manually with MEGA4 (Tamura et al. 2007). For each dataset, Maximum Parsimony (MP) and Maximum Likelihood (ML) analyses were performed.

PAUP v. 4.0 b10 (Swofford 2003) was used for MP analyses, with gaps treated as the fifth character. Uninformative characters were excluded and informative characters were unordered and of equal weight with 1000 random addition replicates. The most parsimonious trees were obtained using the heuristic search function with stepwise addition, tree bisection and reconstruction branch swapping. Maxtrees were set to 5000 and zero-length branches were collapsed. A bootstrap analysis (50% majority rule, 1000 replicates) was undertaken to determine statistical support for the internal nodes in the trees. Tree length (TL), consistency index (CI), retention index (RI) and homoplasy index (HI) were used to assess the trees (Hillis and Huelsenbeck 1992).

PhyML v. 3.1 was used for the ML analyses for each dataset (Guindon and Gascuel 2003). The best nucleotide substitution model for each dataset was determined using the software package jModeltest v. 1.2.5 (Posada 2008). In PhyML, the maximum number of retained trees was set to 1000 and nodal support was determined by non-parametric bootstrapping with 1000 replicates. The phylogenetic trees for both MP and ML analyses were viewed in MEGA4 and edited in Microsoft Office PowerPoint version 2013.

### Morphology

Isolates of the Cryphonectriaceae were grown at 25 °C on 2% malt extract agar (MEA: 20 g/l malt extract and 15 g/l agar, Biolab, Midrand, South Africa and 1000 ml sterile deionised water) containing 0.05 g/l of the antibiotic streptomycin sulphate (Sigma-Aldrich, Steinheim, Germany). Where no sporulation was obtained on agar media, six isolates, representing the putative new species, were inoculated on water agar medium on to which ~ 5 cm long sterilised *Eucalyptus* stem sections had been placed. These were kept at room temperature (~ 25 °C) in the dark until fruiting structures were observed. For each new taxon, micro-morphological structures were studied using Nikon microscopes (Eclipse N*i*, SMZ18, Tokyo, Japan) and a mounted Nikon DS-Ri2 camera. The structures were initially mounted in water, later being replaced with 85% lactic acid on glass microscope slides. In order to study the morphology of fruiting structures and stromatic tissues, pieces of bark, bearing fungal fruiting structures, were mounted on discs in Leica Tissue Freezing Medium and dissected to 12–16 µm thickness using a Leica CM1520 cryostat (Wetzler, Germany). The cut sections were mounted in 85% lactic acid for observation. Whenever available, up to 50 measurements of characteristic features were made for isolates chosen to represent the types of putative new species. Measurements were recorded as minimum-maximum, except for spore dimensions for which supplementary information (mean ± standard deviation) was added.

Growth in culture was examined for two isolates of each putative new species identified. The protocols used to assess growth in culture were the same as those described by Chen et al. (2011). The growth rate at optimum temperature was repeated twice for ex-holotype isolates and the average was presented.

### Pathogenicity tests

*Syzygium jambos* seeds were collected from a garden in Pretoria, South Africa and germinated to produce seedlings for artificial inoculation studies under quarantine greenhouse conditions. These seedlings were grown for one year, until their stem diameters had reached at least 0.5 cm. Ten seedlings (~ 0.5–1 cm diam. × 30 cm high), were inoculated with each test strain and ten seedlings of the same size were inoculated with a sterile agar disc to serve as controls. Inoculations were made using the same technique as that described by Chen et al. (2011). Four weeks (28 days) after inoculation, the lengths of the lesions in the cambium on each plant were measured. The JMP version 5.0 of SAS software (SAS Institute Inc. 2002) was used for statistical analysis of the lesion length data. One way ANOVA was used to test statistical differences between the means of the lesion lengths. Re-isolations were made from the lesions to confirm that they had resulted from the effects of the inoculated fungi.

### Genetic diversity of *Chr. deuterocubensis* isolates

The genetic diversity of the most commonly encountered and globally important species in the Cryphonectriaceae from Myrtales on the Hawaiian Islands was analysed using microsatellite markers. DNA was extracted from all isolates of freshly-prepared cultures using PrepMan Ultra Sample Preparation Reagent (Applied Biosystems, California, USA), following the manufacturer’s instructions. A set of ten microsatellite markers (Suppl. material 1: Table S1), that had been developed and used in previous studies (Van der Merwe et al. 2003, 2010), was tested on ten randomly-selected isolates. The PCR reaction mixes and thermal cycling were the same as those described by Van der Merwe et al. (2003, 2010). PCR aliquots of 5 µl were pre-stained with GelRed Nucleic Acid Gel stain (Biotium, Hayward, USA) and amplicons were separated on 1% (w/v) agarose gels and visualised under UV light to confirm successful amplification. Primer pairs that did not amplify the target loci successfully after several repetitions were discarded. Those that were successful were used to amplify target loci from all the isolates of the available population.

PCR products for each isolate were multiplexed for GeneScan analysis. The composition of each sample mix was the same as that described by Kamgan Nkuekam et al. (2009). Sample mixes were separated on a 36-cm capillary column with POPTM4 polymer on an ABI Prism 3500 sequencer (Perkin-Elmer, Warrington, UK). Allele sizes were determined by comparing the mobility of the PCR products with that of a LIZ 500 size standard (Applied Biosystems, Foster City, California). Microsatellite size data were analysed using the software GeneMapper version 3.0 (Applied Biosystems, Foster City, California).

The allele size for each of the seven loci was scored for each isolate from the collection. These data were used to generate a multilocus haplotype profile for each isolate. Isolates that had identical alleles for each of the seven loci were treated as clones. The frequency of each allele within the collection was calculated by taking the number of times the allele was present in the population and dividing it by the population sample size. This was then used to calculate gene diversity using the formula $H=1-\sum_{k}x_{k}^{2}$ (Nei 1973), where *x_k_* is the frequency of the *k*^th^ allele.

## Results

### Collection of samples and fungal isolation

A total of 139 Cryphonectriaceae isolates were obtained from 106 trees sampled on three Hawaiian Islands (Table 1). Trees, from which the fungi were obtained, included a single specimen of the native species, Ohia (*Metrosideros polymorpha*) and multiple specimens of four non-native Myrtaceae hosts, including an unknown *Metrosideros* sp., *Psidium cattleianum*, *S. cumini* and *S. jambos*. The majority of trees sampled were those of *S. jambos* (66 trees), since this was the main tree of focus in the study and it also displayed the most evident examples of rust infection and death at the time of the survey. Samples were obtained from dead sapling trees (~ 0.5 cm or more diameter) or from older dying/dead trees and cankers on living trees. In the case of *M. polymorpha*, a species of Cryphonectriaceae was obtained from the surface of a single cut stump. On older trees, signs and symptoms of the Cryphonectriaceae could be found on dead branches and stem cankers, including on trees with no obvious infection by the myrtle rust pathogen.

**Table 1.** List of Cryphonectriaceae isolates collected during surveys in Hawaii and sequenced in the study.

Species	Island	Hosts	Number of Trees	Number of Strains
*Chrysoporthe deuterocubensis*	O’ahu	*Syzygium jambos*	18	19
˝	˝	*Syzygium cumini*	3	3
˝	˝	*Syzygium* sp.	11	11
˝	˝	*Psidium cattleianum*	9	12
˝	Hawaii	*S. jambos*	28	38
˝	˝	*Syzygium* sp.	1	1
˝	˝	*Metrosideros polymorpha*	1	1
˝	Maui	*S. jambos*	7	8
*Microthia havanensis*	O’ahu	*P. cattleianum*	5	7
˝	˝	*S. cumini*	1	1
˝	Hawaii	*P. cattleianum*	1	1
˝	˝	*S. jambos*	1	1
*Celoporthe hauoliensis*	Maui	*S. jambos*	4	8
˝	Hawaii	*S. jambos*	2	4
˝	˝	*P. cattleianum*	1	2
*Cel. hawaiiensis*	O’ahu	*P. cattleianum*	4	6
˝	˝	*S. jambos*	3	4
˝	˝	*Syzygium* sp.	1	1
*Cel. paradisiaca*	O’ahu	*P. cattleianum*	1	4
˝	˝	*S. jambos*	2	3
˝	˝	*Syzygium* sp.	1	3
˝	Hawaii	*S. jambos*	1	1

### Phylogenetic analyses

For the isolates selected for sequencing, the PCR fragments were approximately 550, 450 and 260 bp for the ITS, *BT1* and *TEF1* regions, respectively. All sequences obtained in this study were deposited in GenBank (Table 2). The alignments of each of the datasets were deposited in TreeBASE (http://treebase.org, study ID: S19035). The number of taxa and characters in each of the datasets and a summary of the most important parameters applied in the Maximum Parsimony (MP) and Maximum Likelihood (ML) analyses are presented in Suppl. material 2: Table S2.

**Table 2.** List of isolates and their GenBank accession numbers used for DNA sequence comparisons.

Identity	Isolate No.^1,2^	Host	Location	Collector	GenBank accession no.			Reference
					ITS	*BT1*	*TEF1*	
*Amphilogia gyrosa*	CMW10469T	*Elaeocarpus dentatus*	New Zealand	G.J. Samuels	AF452111	AF525707	N/A^3^	Gryzenhout et al. (2005a, 2006c)
	CMW10470	*Ela. dentatus*	New Zealand	G.J. Samuels	AF452112	AF525708	N/A	Gryzenhout et al. (2005a, 2006c)
*Aurantioporthe corni*	CMW10526	*Cornus alternifolia*	USA	S. Redlin	DQ120762	DQ120769	N/A	Gryzenhout et al. (2006c)
	MES1001	N/A	USA	W. Cullina	KF495039	KF495069	N/A	Beier et al. (2015)
	CTS1001	N/A	USA	K. Kitka	KF495033	KF495063	N/A	Beier et al. (2015)
*Aurantiosacculus acutatus*	CBS132181T	*Eucalyptus viminalis*	Australia	B.A. Summerell & P. Summerell	JQ685514	N/A	N/A	Crous et al. (2012)
*Aurantiosacculus eucalyptorum*	CBS130826T	*Euc. globulus*	Australia	C. Mohammed & M. Glen	JQ685515	N/A	N/A	Crous et al. (2012)
*Aurapex penicillata*	CMW10030T	*Miconia theaezans*	Colombia	C.A. Rodas	AY214311	AY214239	N/A	Gryzenhout et al. (2006b, 2009)
	CMW10035	*Mic. theaezans*	Colombia	C.A. Rodas	AY214313	AY214241	N/A	Gryzenhout et al. (2006b, 2009)
*Aurifilum marmelostoma*	CMW28285T	*Terminalia mantaly*	Cameroon	D. Begoude & J. Roux	FJ882855	FJ900585	N/A	Begoude et al. (2010), Vermeulen et al. (2011)
	CMW28288	*Ter. ivorensis*	Cameroon	D. Begoude & J. Roux	FJ882856	FJ900586	N/A	Begoude et al. (2010), Vermeulen et al. (2011)
*Aurifilum terminali*	CSF10757T	*Ter. neotaliala*	China	S.F. Chen & W. Wang	MN199837	MN258775	MN258780	Wang et al. (2020)
	CSF10762	*Ter. neotaliala*	China	S.F. Chen & W. Wang	MN199838	MN258776	MN258781	Wang et al. (2020)
*Capillaureum caryovora*	CBL02T	*Caryocar brasiliense*	Brazil	M.E. Soares de Oliveira &M.A. Ferreira	MG192094	MG211827	N/A	Ferreira et al. (2019)
	CBL06	*Car. brasiliense*	Brazil	M.E. Soares de Oliveira &M.A. Ferreira	MG192096	MG211829	N/A	Ferreira et al. (2019)
*Celoporthe borbonica*	CMW44128T	*Tibouchina grandiflora*	La Réunion	M.J. Wingfield	MG585741	MG585725	N/A	Ali et al. (2018)
	CMW44139	*Tib. grandiflora*	La Réunion	M.J. Wingfield	MG585742	MG585726	N/A	Ali et al. (2018)
*Celoporthe cerciana*	CERC9128T	*Eucalyptus* hybrid tree 4	China, GuangDong	S.F. Chen	MH084352	MH084382	MH084442	Wang et al. (2018)
	CERC9125	*Eucalyptus* hybrid tree 1	China, GuangDong	S.F. Chen	MH084349	MH084379	MH084439	Wang et al. (2018)
*Celoporthe dispersa*	CMW9976T	*S. cordatum*	South Africa	M. Gryzenhout	DQ267130	DQ267136	HQ730840	Nakabonge et al. (2006), Chen et al. (2011)
	CMW9978	*S. cordatum*	South Africa	M. Gryzenhout	AY214316	DQ267135	HQ730841	Nakabonge et al. (2006), Chen et al. (2011)
*Celoporthe eucalypti*	CMW26900	*Eucalyptus* cloneEC48	China	X.D. Zhou & S.F. Chen	HQ730836	HQ730816	HQ730849	Chen et al. (2011)
	CMW26908T	*Eucalyptus* cloneEC48	China	X.D. Zhou & S.F. Chen	HQ730837	HQ730817	HQ730850	Chen et al. (2011)
*Celoporthe fontana*	CMW29375	*S. guineense*	Zambia	M. Vermeulen & J. Roux	GU726940	GU726952	JQ824073	Vermeulen et al. (2013)
	CMW29376T	*S. guineense*	Zambia	M. Vermeulen & J Roux	GU726941	GU726953	JQ824074	Vermeulen et al. (2013)
*Celoporthe guangdongensis*	CMW12750T	*Eucalyptus* sp.	China	T.I. Burgess	HQ730830	HQ730810	HQ730843	Chen et al. (2011)
*Celoporthe hauoliensis*	**CMW38373** ^5^	*S. jambos*	Hawaii	J. Roux	KJ027503	KJ027479	KJ027488	This study
	**CMW38389**T^5^	*P. cattleianum*	Hawaii	J. Roux	KJ027502	KJ027478	KJ027487	This study
	**CMW38546**	*Syzygium* sp.	Hawaii	J. Roux	KJ027504	KJ027480	KJ027489	This study
*Celoporthe hawaiiensis*	**CMW38553** ^5^	*S. jambos*	Hawaii	J. Roux	KJ027500	KJ027476	KJ027485	This study
	**CMW38582**	*S. jambos*	Hawaii	J. Roux	KJ027501	KJ027477	KJ027486	This study
	**CMW38610**T^5^	*S. jambos*	Hawaii	J. Roux	KJ027499	KJ027475	KJ027484	This study
*Celoporthe indonesiensis*	CMW10781T	*S. aromaticum*	Indonesia	M.J. Wingfield	AY084009	AY084033	HQ730842	Myburg et al. (2003), Chen et al. (2011)
*Celoporthe paradisiaca*	**CWM38360**T^4,5^	*Psidium cattleianum*	Hawaii	J. Roux	KJ027498	KJ027474	KJ027483	This study
	**CMW38368**	*Syzygium jambos*	Hawaii	J. Roux	KJ027496	KJ027472	KJ027481	This study
	**CMW38384**	*S. jambos*	Hawaii	J. Roux	KJ027497	KJ027473	KJ027482	This study
*Celoporthe syzygii*	CMW34023T	*S. cumini*	China	S.F. Chen	HQ730831	HQ730811	HQ730844	Chen et al. (2011)
	CMW24912	*S. cumini*	China	M.J. Wingfield & X.D. Zhou	HQ730833	HQ730813	HQ730846	Chen et al. (2011)
*Celoporthe tibouchineae*	CMW44126T	*Tib. grandiflora*	La Réunion	M.J. Wingfield	MG585747	MG585731	N/A	Ali et al. (2018)
	CMW44127	*Tib. grandiflora*	La Réunion	M.J. Wingfield	MG585748	MG585732	N/A	Ali et al. (2018)
*Celoporthe woodiana*	CMW13936T	*Tib. granulosa*	South Africa	M. Gryzenhout	DQ267131	DQ267137	JQ824071	Vermeulen et al. (2013)
	CMW13937	*Tib. granulosa*	South Africa	M. Gryzenhout	DQ267132	DQ267138	JQ824072	Vermeulen et al. (2013)
*Chrysomorbus lagerstroemiae*	CERC8780	*Lagerstroemia speciosa*	China	J. Roux & S.F. Chen	KY929330	KY929350	N/A	Chen et al. (2018)
	CERC8810T	*L. speciosa*	China	S.F. Chen	KY929338	KY929358	N/A	Chen et al. (2018)
*Chrysoporthe austroafricana*	CMW62	*Euc. grandis*	South Africa	M.J. Wingfield	AF292041	AF273063	N/A	Myburg et al. (2002b), Gryzenhout et al. (2006c)
	CMW9327	*Tib. granulosa*	South Africa	J. Roux	AF273473	AF273060	N/A	Myburg et al. (2002a)
	CMW2113T	*Euc. grandis*	South Africa	M.J. Wingfield	AF046892	AF273067	N/A	Myburg et al. (1999, 2002b)
*Chrysoporthe cubensis*	CMW10453	*Euc. saligna*	Democratic Republic of the Congo	N/A	AY063476	AY063478	N/A	Castlebury et al. (2002), Gryzenhout et al. (2004)
	CMW8758	*Eucalyptus* sp.	Venezuela	M.J. Wingfield	AF046898	AF273068	N/A	Myburg et al. (2002b), Gryzenhout et al. (2006c)
	CMW10669	*Eucalyptus* sp.	Republic of the Congo	J. Roux	AF535122	AF535124	N/A	Gryzenhout et al. (2004)
	CMW10639	*Euc. grandis*	Colombia	C.A. Rodas	AY263421	AY263419	N/A	Gryzenhout et al. (2004)
*Chrysoporthe deuterocubensis*	CMW11290	*Eucalyptus* sp.	Indonesia	M.J. Wingfield	AY214304	AY214232	N/A	Gryzenhout et al. (2004)
	CMW8651	*S. aromaticum*	Indonesia	M.J. Wingfield	AY084002	AY084026	N/A	Myburg et al. (2003)
	**CMW38375** ^5^	*P. cattleianum*	Hawaii	J. Roux	KJ027490	KJ027466	N/A	This study
	**CMW38549** ^5^	*S. jambos*	Hawaii	J. Roux	KJ027491	KJ027467	N/A	This study
	**CMW38565**	*Metrosideros polymorpha*	Hawaii	J. Roux	KJ027492	KJ027468	N/A	This study
*Chrysoporthe doradensis*	CMW11287T	*Euc. grandis*	Ecuador	M.J. Wingfield	AY214289	AY214217	N/A	Gryzenhout et al. (2005b)
	CMW11286	*Euc. grandis*	Ecuador	M.J. Wingfield	AY214290	AY214218	N/A	Gryzenhout et al. (2005b)
*Chrysoporthe hodgesiana*	CMW10625	*Mic. theaezans*	Colombia	C.A. Rodas	AY956970	AY956979	N/A	Rodas et al. (2005)
	CMW9995	*Tib. semidecandra*	Colombia	R. Arbelaez	AY956969	AY956977	N/A	Rodas et al. (2005)
	CMW10641T= CBS115854	*Tib. semidecandra*	Colombia	R. Arbelaez	AY692322	AY692326	N/A	Gryzenhout et al. (2004)
*Chrysoporthe inopina*	CMW12727T	*Tib. lepidota*	Colombia	R. Arbelaez	DQ368777	DQ368806	N/A	Gryzenhout et al. (2006d)
	CMW12729	*Tib. lepidota*	Colombia	R. Arbelaez	DQ368778	DQ368808	N/A	Gryzenhout et al. (2006d)
*Chrysoporthe syzygiicola*	CMW29940T= CBS124488	*S. guineense*	Zambia	D. Chungu & J. Roux	FJ655005	FJ805230	N/A	Chungu et al. (2010)
	CMW29942= CBS124490	*S. guineense*	Zambia	D. Chungu & J. Roux	FJ655007	FJ805232	N/A	Chungu et al. (2010)
*Chrysoporthe zambiensis*	CMW29928T= CBS124503	*Euc. grandis*	Zambia	D. Chungu & J. Roux	FJ655002	FJ858709	N/A	Chungu et al. (2010)
	CMW29930= CBS124502	*Euc. grandis*	Zambia	D. Chungu & J. Roux	FJ655004	FJ858711	N/A	Chungu et al. (2010)
*Corticimorbus sinomyrti*	CERC3629T	*Rhodomyrtus tomentosa*	China	S.F. Chen & G.Q. Li	KT167169	KT167189	N/A	Chen et al. (2016)
	CERC3631	*Rho. tomentosa*	China	S.F. Chen & G.Q. Li	KT167170	KT167190	N/A	Chen et al. (2016)
*Cryphonectria parasitica*	CMW7048	*Q. virginiana*	USA	R.J. Stipes	AF368330	AF273076	N/A	Venter et al. (2002), Gryzenhout et al. (2006c)
	CMW13749	*Cas. mollisima*	Japan	N/A	AY697927	AY697943	N/A	Myburg et al. (2004)
*Cryphonectria quercus*	CFCC52138T	Q. aliena var. acuteserrata	China, ShaanXi	N. Jiang	MG866024	MG896115	N/A	Jiang et al. (2018)
	CFCC52139	Q. aliena var. acuteserrata	China, ShaanXi	N. Jiang	MG866025	MG896116	N/A	Jiang et al. (2018)
*Cryphonectria radicalis*	CMW10455	*Q. suber*	Italy	A. Biraghi	AF452113	AF525705	N/A	Gryzenhout et al. (2006c)
	CMW10477	*Q. suber*	Italy	A. Biraghi	AF368328	AF368347	N/A	Venter et al. (2002), Gryzenhout et al. (2006c)
*Cryptometrion aestuescens*	CMW18790	*Euc. grandis*	Indonesia	M.J. Wingfield	GQ369458	GQ369455	N/A	Gryzenhout et al. (2010), Vermeulen et al. (2011)
	CMW18793	*Euc. grandis*	Indonesia	M.J. Wingfield	GQ369459	GQ369456	N/A	Gryzenhout et al. (2010), Vermeulen et al. (2011)
	CMW28535T= CBS124009	*Euc. grandis*	North Sumatra, Indonesia	M.J. Wingfield	GQ369457	GQ369454	N/A	Gryzenhout et al. (2010)
*Diversimorbus metrosiderotis*	CMW37321	*Metrosideros angustifolia*	South Africa	J. Roux	JQ862870	JQ862911	N/A	Chen et al. (2013b)
	CMW37322T	*Met. angustifolia*	South Africa	J. Roux	JQ862871	JQ862912	N/A	Chen et al. (2013b)
*Endothia gyrosa*	CMW2091	*Q. palustris*	USA	R.J. Stipes	AF368325	AF368337	N/A	Venter et al. (2002), Gryzenhout et al. (2006c)
	CMW10442	*Q. palustris*	USA	R.J. Stipes	AF368326	AF368339	N/A	Venter et al. (2002), Gryzenhout et al. (2006c)
*Holocryphia capensis*	CMW37887T	*Met. angustifolia*	South Africa	J. Roux, S.F. Chen & F. Roets	JQ862854	JQ862895	JQ863051	Chen et al. (2013b)
	CMW37329	*Met. angustifolia*	South Africa	J. Roux & S.F. Chen	JQ862859	JQ862900	JQ863056	Chen et al. (2013b)
*Holocryphia eucalypti*	CMW7033T	*Euc. grandis*	South Africa	M. Venter	JQ862837	JQ862878	JQ863034	Chen et al. (2013b)
	CMW7035	*Euc. saligna*	South Africa	M. Venter	JQ862838	JQ862879	JQ863035	Chen et al. (2013b)
*Holocryphia gleniana*	CMW37334T	*Met. angustifolia*	South Africa	J. Roux & S.F. Chen	JQ862834	JQ862875	JQ863031	Chen et al. (2013b)
	CMW37335	*Met. angustifolia*	South Africa	J. Roux & S.F. Chen	JQ862835	JQ862876	JQ863032	Chen et al. (2013b)
*Holocryphia mzansi*	CMW37337T	*Met. angustifolia*	South Africa	J. Roux & S.F. Chen	JQ862841	JQ862882	JQ863038	Chen et al. (2013b)
	CMW37338	*Met. angustifolia*	South Africa	J. Roux & S.F. Chen	JQ862842	JQ862883	JQ863039	Chen et al. (2013b)
*Holocryphia* sp.	CMW6246	*Tib. granulosa*	Australia	M.J. Wingfield	JQ862845	JQ862886	JQ863042	Chen et al. (2013b)
	CMW10015	*Euc. fastigata*	New Zealand	R.J. van Boven	JQ862849	JQ862890	JQ863046	Chen et al. (2013b)
*Immersiporthe knoxdaviesiana*	CMW37314T	*Rapanea melanophloeos*	South Africa	M.J. Wingfield & J. Roux	JQ862765	JQ862785	N/A	Chen et al. (2013a)
	CMW37315	*Rap. melanophloeos*	South Africa	M.J. Wingfield & J. Roux	JQ862766	JQ862786	N/A	Chen et al. (2013a)
*Latruncella aurorae*	CMW28274	*Galpinia transvaalica*	Swaziland	J. Roux	GU726946	GU726958	N/A	Vermeulen et al. (2011)
	CMW28276T	*G. transvaalica*	Swaziland	J. Roux	GU726947	GU726959	N/A	Vermeulen et al. (2011), Chen et al. (2011)
	CMW28275	*G. transvaalica*	Swaziland	J. Roux	HQ171209	HQ171207	N/A	Vermeulen et al. (2011)
*Luteocirrhus shearii*	CBS130775	*Banksia baxteri*	Australia	C. Crane	KC197024	KC197015	N/A	Crane and Burgess (2013)
	CBS130776T	*B. baxteri*	Australia	C. Crane	KC197021	KC197012	N/A	Crane and Burgess (2013)
*Microthia havanensis*	CMW11301	*Myr. faya*	Azores	C.S. Hodges & D.E. Gardner	AY214323	AY214251	N/A	Gryzenhout et al. (2006a)
*Microthia havanensis*	CMW14550	*E. saligna*	Mexico	C.S. Hodges	DQ368735	DQ368741	N/A	Gryzenhout et al. (2006a)
	**CMW38563** ^e^	*S. jambos*	Hawaii	J. Roux	KJ027493	KJ027469	N/A	This study
	**CMW38367**	*P. cattleianum*	Hawaii	J. Roux	KJ027495	KJ027471	N/A	This study
	**CMW38585** ^e^	*S. jambos*	Hawaii	J. Roux	KJ027494	KJ027470	N/A	This study
*Myrtonectria myrtacearum*	CMW46433T	*Heteropyxis natalensis*	South Africa	D.B. Ali & J. Roux	MG585736	MG585720	N/A	Ali et al. (2018)
	CMW46435	*S. cordatum*	South Africa	D.B. Ali & J. Roux	MG585737	MG585721	N/A	Ali et al. (2018)
*Parvosmorbus eucalypti*	CSF2061T	*E. urophylla × E. grandis* hybrid clone	China	S.F. Chen & G.Q. Li	MN258788	MN258816	MN258830	Wang et al. (2020)
	CSF8777	*E. urophylla* hybrid clone	China	J.Roux & S.F. Chen	MN258794	MN258822	MN258836	Wang et al. (2020)
*Parvosmorbus guangdongensis*	CSF10460T	*E. urophylla* hybrid clone	China	S.F. Chen & W. Wang	MN258799	MN258827	MN258841	Wang et al. (2020)
	CSF10738	*E. grandis* hybrid clone	China	S.F. Chen & W. Wang	MN258800	MN258828	MN258842	Wang et al. (2020)
*Rostraureum tropicale*	CMW9972	*Terminalia ivorensis*	Ecuador	M.J. Wingfield	AY167436	AY167426	N/A	Gryzenhout et al. (2005c, 2006c)
	CMW10796T	*Ter. ivorensis*	Ecuador	M.J. Wingfield	AY167438	AY167428	N/A	Gryzenhout et al. (2005c)
	CMW9971	*Ter. ivorensis*	Ecuador	M.J. Wingfield	AY167435	AY167425	N/A	Gryzenhout et al. (2005c)
*Ursicollum fallax*	CMW18119T	*Coccoloba uvifera*	USA	C.S. Hodges	DQ368755	DQ368758	N/A	Gryzenhout et al. (2006a, 2009)
	CMW18115	*Coc. uvifera*	USA	C.S. Hodges	DQ368756	DQ368760	N/A	Gryzenhout et al. (2006a)
*Diaporthe ambigua*	CMW5587	*Malus domestica*	South Africa	W.A. Smit	AF543818	AF543820	N/A	Gryzenhout et al. (2006a)
	CMW5288	*M. domestica*	South Africa	W.A. Smit	AF543817	AF543819	N/A	Gryzenhout et al. (2006a)

^1^ Designation of isolates and culture collections: ATCC = American Type Culture Collection, Manassas, USA; CBL represent isolates in Ferreira et al. (2019); CBS = Westerdijk Fungal Biodiversity Institute, Utrecht, Netherlands; CERC = China Eucalypt Research Centre (CERC), Chinese Academy of Forestry (CAF), ZhanJiang, GuangDong, China; CFCC = China Forestry Culture Collection Center, Beijing, China; CMW = Tree Protection Co-operative Program, Forestry and Agricultural Biotechnology Institute, University of Pretoria, South Africa; CSF = Culture Collection from Southern Forests (CSF), China Eucalypt Research Centre, Chinese Academy of Forestry, ZhanJiang, GuangDong, China; MES, CTS represent isolates in Beier et al. (2015). ^2^ ‘T’ following isolate number means isolates are ex-type or from samples that have been linked morphologically to type material of the species. ^3^ N/A = not available. ^4^ Isolates identified in this study are in bold font type. ^5^ Isolates used for inoculations.

**Figure 1.**
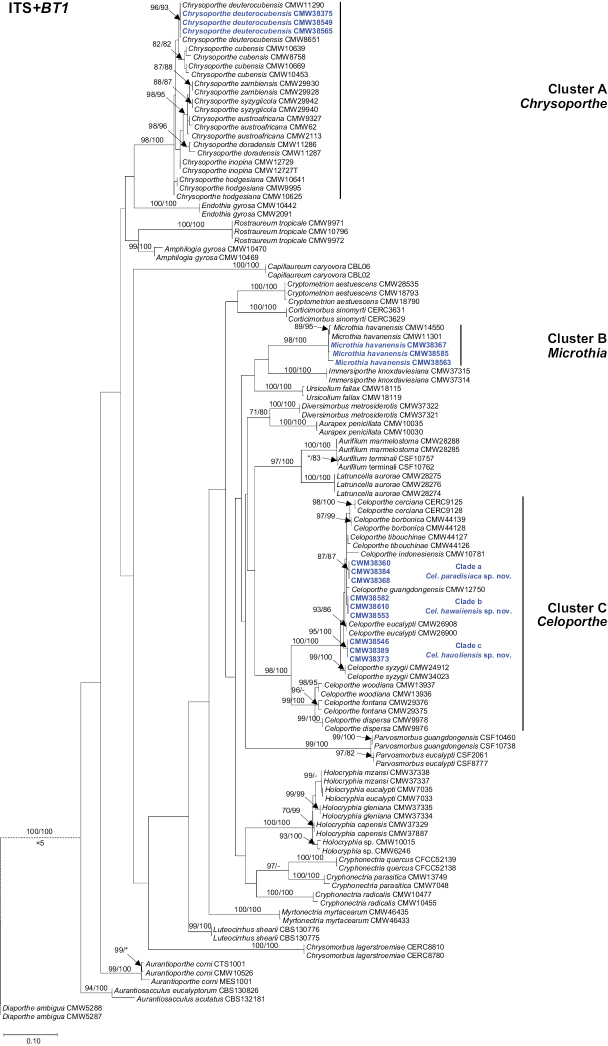
Phylogenetic trees based on Maximum Likelihood (ML) analyses of a combined DNA sequence dataset of ITS and *BT1* regions for various genera in the Diaporthales. Bootstrap values ≥ 70% for ML and MP (maximum parsimony) analyses are presented at branches as follows: ML/MP. Bootstrap values lower than 70% are marked with * and absent analysis values are marked with –. Isolates collected in this study are in boldface and blue. *Diaporthe ambigua* (CMW5287 and CMW5588) (Diaporthaceae) was used as the outgroup taxon.

For the ITS and *BT1* datasets, the PHT generated a value of P = 0.001, indicating that the accuracy of the combined data had not suffered relative to the individual partitions (Cunningham 1997). Sequences of the two regions were combined for analyses. For each of the ITS, *BT1* and ITS+*BT1* datasets, the ML and MP analyses generated trees with generally consistent topologies and phylogenetic relationships amongst taxa. Based on the phylogenetic analyses of the ITS, *BT1* and combined datasets, the isolates obtained in this study were grouped in three Clusters, referred to as Clusters A–C (Fig. 1; ITS and *BT1* trees not presented). Isolates in Cluster A grouped in the genus *Chrysoporthe* and they all resided in the same phylogenetic clade as *Chrysoporthe deuterocubensis*. Isolates in Cluster B grouped in the genus *Microthia* and were phylogenetically closely related to *Microthia havanensis*. Isolates in Cluster C grouped with species of *Celoporthe*. They formed three distinct Clades (Clades a–c) within *Celoporthe* based on the ITS+*BT1* tree (Fig. 1).

**Figure 2.**
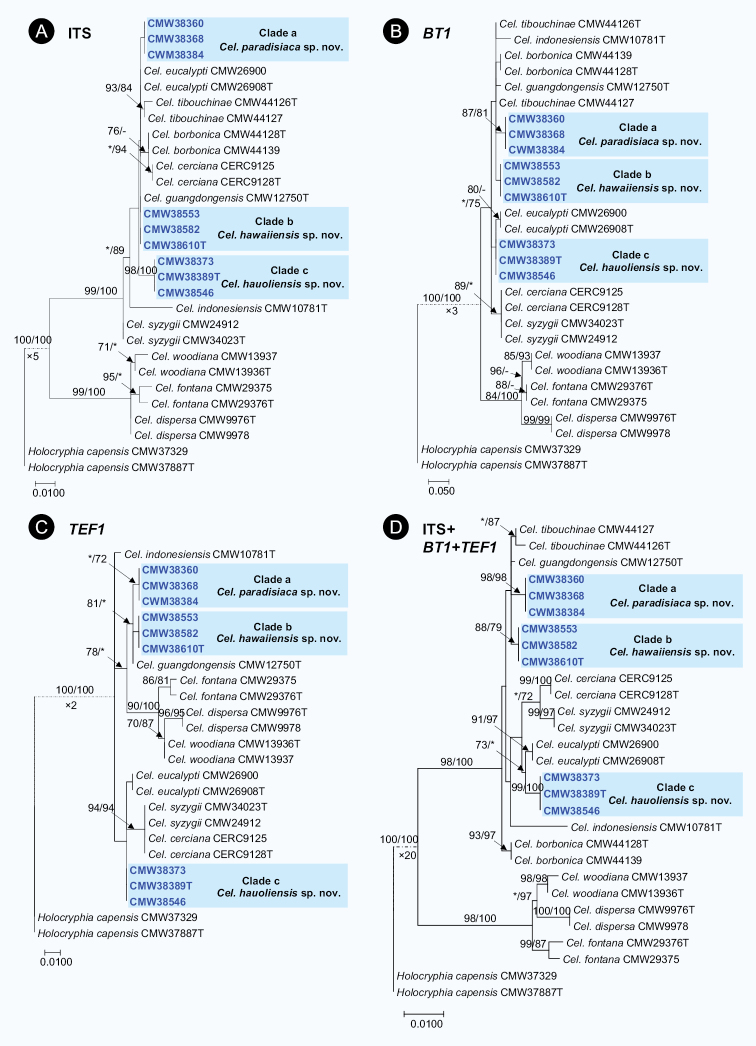
Phylogenetic trees, based on Maximum Likelihood (ML) analyses for species in *Celoporthe***A**ITS region **B***BT1* gene region **C***TEF1* gene region **D** combined ITS, *BT1* and *TEF1* regions. Bootstrap values ≥ 70% for ML and MP (maximum parsimony) analyses are presented at branches as follows: ML/MP. Bootstrap values lower than 70% are marked with * and absent analysis values are marked with –. Isolates collected in this study are in boldface and blue. *Holocryphia capensis* (CMW37329 and CMW37887) was used as the outgroup taxon.

In the ITS, *BT1* and *TEF1* datasets for *Celoporthe* isolates, the PHT generated a value of P = 0.001, showing that the accuracy of the combined data were unaffected relative to the individual partitions (Cunningham 1997) and the three gene regions were thus combined in the analyses. Other than the ITS tree (Fig. 2A), Hawaiian isolates formed distinct lineages (Clades a–c) that differentiated them from other *Celoporthe* species (Fig. 2B–D). In the combined analyses of ITS, *BT1* and *TEF1* gene sequences, isolates in each of Clades a, b and c formed independent lineages, supported by high bootstrap values (Clade a: ML/MP = 98%/98%; Clade b: ML/MP = 88%/79%; Clade c: ML/MP = 99%/100%) (Fig. 2D). These three clades were consequently recognised as representing three undescribed species. Isolates in Clades a and b were most closely related to *Celoporthe guangdongensis* and those in Clade c were all most closely related to *Cel. eucalypti* and *Cel. cerciana* (Fig. 2D).

### Morphology

Fruiting bodies developed for all six isolates grown on *Eucalyptus* stem sections on water agar after two months of incubation at room temperature. Other than some minor differences, all fungal isolates, obtained in this study, were morphologically similar. This was consistent with the fact that fungi in the Cryphonectriaceae are mostly indistinguishable on artificial media (Gryzenhout et al. 2009).

Colonies on 2% MEA were fluffy and white when young, turning yellow or greenish-grey to greenish when old. The optimal growth temperatures for novel species was 30 °C, at which colonies reached 59–80 mm within 4 days.

### Taxonomy

Based on phylogenetic analyses of sequence data for the three gene regions, three previously unknown Cryphonectriaceae species are recognised from non-native Myrtaceae on the Hawaiian Islands. The three fungi reside in the genus *Celoporthe* and are distinct from described *Celoporthe* species, based on sequence data. Since limited numbers of fruiting bodies were available from the originally-collected plant material for these three species and mostly conidia were obtained under laboratory conditions, they are defined primarily based on multiple gene DNA sequence data. Morphological descriptions are provided for colonies on MEA and fruiting structures produced on *Eucalyptus* stem sections.

#### Celoporthe hauoliensis

Taxon classificationFungiMyrtalesMyrtaceae

Kamgan, Jol. Roux & Marinc. sp. nov.

F105E296-1680-5FBA-A759-EFCD9AE73A21

MycoBank No: 808579

Fig. 3

##### Etymology.

The species name refers to the Hawaiian word for happy, “Hau’oli”, describing the collector’s joy in visiting and discovering Cryphonectriaceae on the Islands.

##### Types.

***Holotype***: USA, Hawaii, O’ahu Island, Pu’u PiaManoa, isolated from bark of *Psidium cattleianum*, 23 July 2012, *J. Roux* (PREM 61309; Ex-type culture CMW38389 = CBS 140640); GenBank accession numbers KJ027502 (ITS), KJ027478 (*BT1*), KJ027487 (*TEF1*). ***Paratypes***: Hawaii, O’ahu Island, Waimea Valley Botanical Gardens, isolated from bark of *Syzygium* sp., 23 July 2012, *J. Roux* (PREM 61310; living culture CMW38546 = CBS 140641). Hawaii, O’ahu Island, Waimea Valley Botanical Garden, isolated from bark of *Syzygium jambos*, July 2012, *J. Roux* (CMW38373).

**Figure 3.**
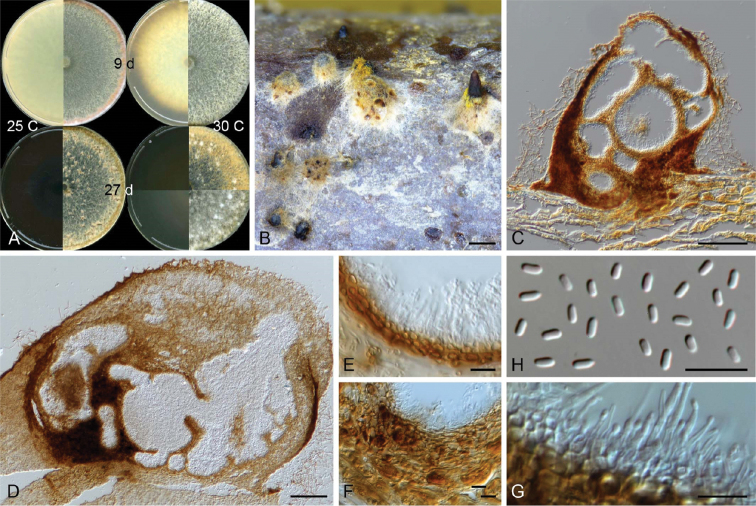
Micrographs of *Celoporthe hauoliensis* sp. nov. (holotype: PREM 61309; ex-holotype CBS 140640 = CMW38389) **A** culture morphology on 2% MEA at 25 °C and 30 °C at 9 and 27 days **B** conidiomata produced on *Eucalyptus* stem sections on water agar **C, D** vertical section of conidioma **E** inner fertile wall of conidioma **F** conidiomatal wall **G** conidiogenous cells **H** conidia. Scale bars: 1 mm (**B**); 100 µm (**C, D**); 10 µm (**E–H**).

##### Sexual morph.

Not observed.

##### Asexual morph.

Formed after two months on *Eucalyptus* stem sections placed on water agar. *Conidiomata* superficial or with base embedded, pulvinate or conical with or without necks, often covered with pigmented hyphae, uni- or multilocular, convoluted, 287–722 µm long, 332–808 µm wide. *Conidiomatal walls* outer- and inter-locular stratum prosenchymatous; inner fertile stratum pseudoparenchymatous, composed of a few layers of brown, flattened, thick-walled cells, 8–26 µm thick. *Paraphyses* present, scarcely observed, 14–26 µm long. *Conidiophores* formed along inner layer of locule, simple or branched, often reduced to conidiogenous cells, 5–21 µm long. *Conidiogenous cells* enteroblastic, lageniform, tapering towards apex, 3–9 × 1–2.5 µm. *Conidia* hyaline, oblong, straight, occasionally curved, aseptate, 3–4 × 1–1.5 (3.09 ± 0.30 × 1.31 ± 0.08) µm.

##### Culture characteristics.

Colonies on 2% MEA, when young showing circular growth with smooth margins, above white with tint of yellow (30 °C) or orange (25 °C) towards the edge of Petri dish, reverse yellow, except for at 30 °C becoming brown towards the edge; with age above becoming brown, except for 30 °C at which each colony showing variable yellow with white mycelial clumps, reverse dark brown at all temperatures; optimal growth at 30 °C (9.4 mm/d), followed by 25 °C (7.9 mm/d) and 20 °C (4.8 mm/d), minimal growth at 35 °C (0.2 mm/d), no growth at 5 °C; mycelia fluffy, density sparce in centre becoming thicker towards the edge.

##### Habitat.

On/in bark of *Psidium cattleianum* and *Syzygium jambos*

##### Distribution.

Hawaii, USA

##### Notes.

*Celoporthe hauoliensis* is morphologically similar to its phylogenetically closest relatives *Cel. eucalypti* and *Cel. cerciana*, but can be differentiated by DNA sequences. In the ITS, *BT1* and *TEF1* datasets, *Cel. hauoliensis* differs from *Cel. eucalypti* by 8, 4 and 4 base pairs and from *Cel. cerciana* by 11, 9 and 6 base pairs, respectively (Tables 3–5).

**Table 3.** Nucleotide differences observed in the ITS region between *Celoporthe hauoliensis*, *Cel. eucalypti* and *Cel. cerciana*.

Species/Isolate No.	ITS ^1^												
	8^2^	61	75	76	80	112	161	162	186	187	193	194	467
*Cel. hauoliensis* CMW383735	T^3^	A	**G**	**C**	**C**	–	–	**C**	T	A	–	**C**	–
*Cel. hauoliensis CMW38389* ^4^	T	A	**G**	**C**	**C**	–	–	**C**	T	A	–	**C**	–
*Cel. hauoliensis* CMW38546	T	A	**G**	**C**	**C**	–	–	**C**	T	A	–	**C**	–
*Cel. eucalypti* CMW26900	–	A	–	T	G	G	**A**	A	T	A	–	A	–
*Cel. eucalypti CMW26908*	–	A	–	T	G	G	**A**	A	T	A	–	A	–
*Cel. cerciana* CERC9125	T	**G**	–	T	G	G	–	A	**A**	**C**	**A**	A	**T**
*Cel. cerciana CERC9128*	T	**G**	–	T	G	G	–	A	**A**	**C**	**A**	A	**T**

^1^ Polymorphic nucleotides occurring only in all isolates are shown, not alleles that partially occur in individuals per phylogenetic group. ^2^ Numerical positions of the nucleotides in the DNA sequence alignments are indicated. ^3^ Fixed polymorphisms for each group are in bold. ^4^ Ex-type isolates are indicated in italic.

**Table 4.** Nucleotide differences observed in the *BT1* gene region between *Celoporthe hauoliensis*, *Cel. eucalypti* and *Cel. cerciana*.

Species/Isolate No.	*BT1* ^1^									
	105^2^	127	130	131	132	182	183	188	191	201
*Cel. hauoliensis* CMW383735	G^3^	C	–	–	–	–	–	–	T	C
*Cel. hauoliensis CMW38389* ^4^	G	C	–	–	–	–	–	–	T	C
*Cel. hauoliensis* CMW38546	G	C	–	–	–	–	–	–	T	C
*Cel. eucalypti* CMW26900	**A**	C	C	T	C	–	–	–	T	C
*Cel. eucalypti CMW26908*	**A**	C	C	T	C	–	–	–	T	C
*Cel. cerciana* CERC9125	G	**T**	C	T	C	**C**	**C**	**C**	**C**	**A**
*Cel. cerciana CERC9128*	G	**T**	C	T	C	**C**	**C**	**C**	**C**	**A**

^1^ Polymorphic nucleotides occurring only in all isolates are shown, not alleles that partially occur in individuals per phylogenetic group. ^2^ Numerical positions of the nucleotides in the DNA sequence alignments are indicated. ^3^ Fixed polymorphisms for each group are in bold. ^4^ Ex-type isolates are indicated in italic.

**Table 5.** Nucleotide differences observed in the *TEF1* gene region between *Celoporthe hauoliensis*, *Cel. eucalypti* and *Cel. cerciana*.

Species/Isolate No.	*TEF1*						
	23^2^	43	44	112	113	114	127
*Cel. hauoliensis* CMW383735	C^3^	G	C	–	–	–	T
*Cel. hauoliensis* CMW38389^4^	C	G	C	–	–	–	T
*Cel. hauoliensis* CMW38546	C	G	C	–	–	–	T
*Cel. eucalypti* CMW26900	**T**	G	C	T	T	T	T
*Cel. eucalypti* CMW26908	**T**	G	C	T	T	T	T
*Cel. cerciana* CERC9125	C	**T**	**T**	T	T	T	**C**
*Cel. cerciana* CERC9128	C	**T**	**T**	T	T	T	**C**

^1^ Polymorphic nucleotides occurring only in all isolates are shown, not alleles that partially occur in individuals per phylogenetic group. ^2^ Numerical positions of the nucleotides in the DNA sequence alignments are indicated. ^3^ Fixed polymorphisms for each group are in bold. ^4^ Ex-type isolates are indicated in italic.

#### Celoporthe hawaiiensis

Taxon classificationFungiMyrtalesMyrtaceae

Kamgan, Jol. Roux & Marinc. sp. nov.

F4E0EDFF-8C8A-5C13-A3EA-9182015161E1

MycoBank No: 808578

Fig. 4

##### Etymology.

The species name refers to the Hawaiian Islands where the holotype was collected.

##### Types.

***Holotype***: USA, Hawaii, Maui Island, Hana Road, 20 miles from Kahului, isolated from bark of *Syzygium jambos*, 30 July 2012, *J. Roux* (PREM61307; Ex-type culture CMW38610 = CBS140642); GenBank accession numbers KJ027499 (ITS), KJ027475 (*BT1*), KJ027484 (*TEF1*). ***Paratypes***: Hawaii, Maui Island, Hana Road, 20 miles from Kahului, isolated from bark of *Syzygium jambos*, 30 July 2012, *J. Roux* (PREM 61308; living culture CMW38582 = CBS140643). Hawaii, Big Island, Rainbow Falls, Hilo, isolated from bark of *Syzygium jambos*, 26 July 2012, *J. Roux* (CMW38553).

**Figure 4.**
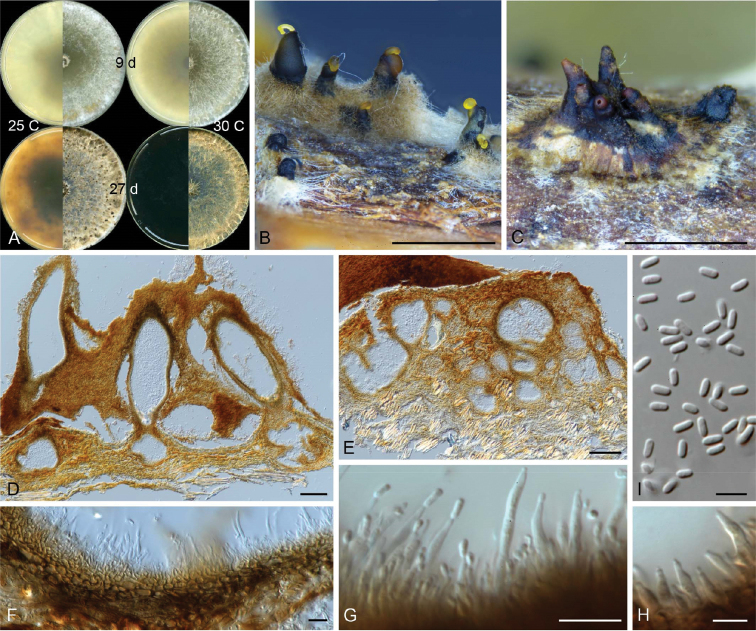
Micrographs of *Celoporthe hawaiiensis* sp. nov. (holotype: PREM 61307, ex-holotype CBS 140642 = CMW38610) **A** culture morphology on 2% MEA at 25 °C and 30 °C at 9 and 27 days **B, C** conidiomata produced on *Eucalyptus* stem sections on water agar **D, E** vertical section of conidioma **F** conidiomatal wall **G, H** conidiogenous cells **I** conidia. Scale bars: 1 mm (**B, C**); 100 µm (**D, E**); 10 µm (**F, G**); 5 µm (**H, I**).

##### Sexual morph.

Not observed.

##### Asexual morph.

Formed after two months on *Eucalyptus* stem sections placed on water agar. *Conidiomata* superficial or with base embedded, single or gregarious, uni- or multilocular, convoluted, base often covered with brown hyphal mass, dark brown to black, pulvinate to conical with or without necks, 450–1814 µm long, 329–1069 µm wide; necks attenuating towards apex, tip of neck paler than body. *Conidiomatal wall* outer-and inter-locular stratum prosenchymatous; inner fertile stratum pseudoparenchymatous, 5–19 µm thick. *Paraphyses* present, cylindrical, tapering towards apex, scarce, 16–29 µm long. *Conidiophores* formed along inner layer of locule, simple or branched, occasionally reduced to conidiogenous cell, 10–26 µm long. *Conidiogenous cells* enteroblastic, lageniform, tapering towards apex, 4–12 × 1–2 µm. *Conidia* hyaline, oblong, aseptate, exuding in yellow droplets or tendril, 2.5–4 × 1–1.5 (3.17 ± 0.27 × 1.27 ± 0.08) µm.

##### Culture characteristics.

Colonies on 2% MEA, when young showing circular growth with smooth margins, above white with yellow tint towards edge (25 °C), reverse pale brown, becoming darker in centre at 25 °C and 30 °C; with age above becoming darker yellow to brown, reverse dark brown, except at 20 °C, 25 °C having yellow with dark brown patches; optimal growth at 30 °C (6.6 mm/d), followed by 25 °C (6.0 mm/d) and 20 °C (4.1 mm/d), minimal growth at 35 °C (0.1 mm/d), growth at 5 °C restricted to mycelial plug; mycelia fluffy, density sparse in centre becoming thicker towards the edge.

##### Habitat.

On/in bark of *Psidium cattleianum*, *Syzygium jambos* and *Syzygium* sp. indet.

##### Distribution.

Hawaii, USA

##### Notes.

*Celoporthe hawaiiensis* is morphologically similar to *Cel. guangdongensis* and *Cel. paradisiaca*, its phylogenetic closest relatives, but can be differentiated by DNA sequences. In the ITS, *BT1* and *TEF1* datasets, *Cel. hawaiiensis* differs from *Cel. guangdongensis* by 3, 3 and 1 base pairs and from *Cel. paradisiaca* by 6, 3 and 3 base pairs, respectively (Tables 6, 7).

**Table 6.** Nucleotide differences observed in the ITS region between *Celoporthe hawaiiensis*, *Cel. guangdongensis* and *Cel. paradisiaca*.

Species/Isolate No.	ITS ^1^							
	56^2^	57	59	98	160	161	193	467
*Cel. paradisiaca CWM38360* ^3^	**A** ^4^	**G**	**A**	–	–	A	**A**	–
*Cel. paradisiaca* CMW38368	**A**	**G**	**A**	–	–	A	**A**	–
*Cel. paradisiaca* CWM38384	**A**	**G**	**A**	–	–	A	**A**	–
*Cel. hawaiiensis* CMW38553	–	–	G	–	–	–	–	T
*Cel. hawaiiensis* CMW38582	–	–	G	–	–	–	–	T
*Cel. hawaiiensis CMW38610* ^3^	–	–	G	–	–	–	–	T
*Cel. guangdongensis CMW12750* ^3^	–	–	G	**C**	**A**	A	–	T

^1^ Polymorphic nucleotides occurring only in all isolates are shown, not alleles that partially occur in individuals per phylogenetic group. ^2^ Numerical positions of the nucleotides in the DNA sequence alignments are indicated. ^3^ Ex-type isolates are indicated in italic. ^4^ Fixed polymorphisms for each group are in bold.

**Table 7.** Nucleotide differences observed in the *BT1* and *TEF1* gene regions between *Celoporthe hawaiiensis*, *Cel. guangdongensis* and *Cel. paradisiaca*.

Species/Isolate No.	*BT1* ^1^					*TEF* ^1^		
	57^2^	131	139	175	272	77	220	222
*Cel. paradisiaca CWM38360* ^3^	C^d^	T	**A**	C	**C**	C	–	**A**
*Cel. paradisiaca* CMW38368	C	T	**A**	C	**C**	C	–	**A**
*Cel. paradisiaca* CWM38384	C	T	**A**	C	**C**	C	–	**A**
*Cel. hawaiiensis* CMW38553	C	**G**	G	C	G	**A**	A	C
*Cel. hawaiiensis* CMW38582	C	**G**	G	C	G	**A**	A	C
*Cel. hawaiiensis CMW38610* ^3^	C	**G**	G	C	G	**A**	A	C
*Cel. guangdongensis CMW12750* ^3^	**T**	T	G	–	G	C	A	C

^1^ Polymorphic nucleotides occurring only in all isolates are shown, not alleles that partially occur in individuals per phylogenetic group. ^2^ Numerical positions of the nucleotides in the DNA sequence alignments are indicated. ^3^ Ex-type isolates are indicated in italic. ^4^ Fixed polymorphisms for each group are in bold.

#### Celoporthe paradisiaca

Taxon classificationFungiMyrtalesMyrtaceae

S.F. Chen & Marinc. sp. nov.

DDD005CD-8120-5690-BA81-1B01144E7F33

MycoBank No: 836918

Fig. 5

##### Etymology.

The species name refers to the fact that Hawaii, where the holotype of this fungus was collected, is regarded as a paradise by travellers.

##### Types.

***Holotype***: USA, Hawaii, O’ahu Island, Ho’omaluhia, isolated from bark of *Psidium cattleianum*, 24 July 2012, *J. Roux* (PREM 63205; Ex-type culture CMW38360 = CBS 147169); GenBank accession numbers KJ027498 (ITS), KJ027474 (*BT1*), KJ027483 (*TEF1*). ***Paratype***: Hawaii, O’ahu Island, Waimea Valley Botanical Gardens, isolated from bark of *Syzygium jambos*, 23 July 2012, *J. Roux* (PREM 63206; living culture CMW38368 = CBS 147170).

**Figure 5.**
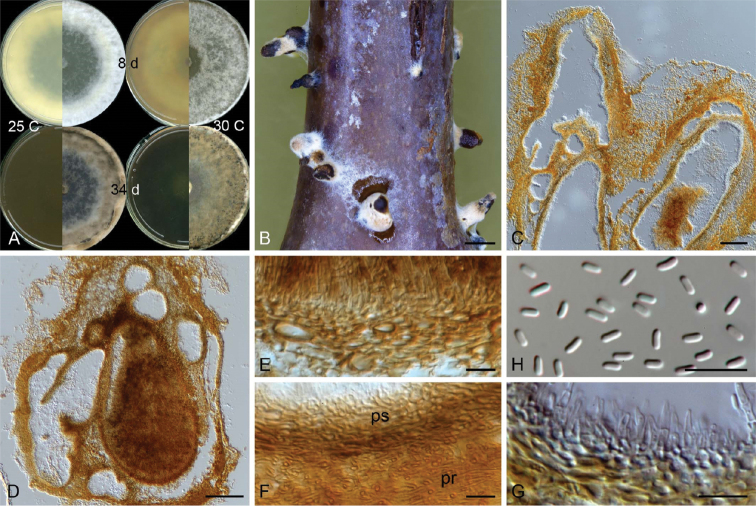
Micrographs of *Celoporthe paradisiaca* sp. nov. (holotype: PREM 63205, ex-holotype CBS 147169 = CMW38360) **A** culture morphology on 2% MEA at 25 °C and 30 °C at 8 and 34 days **B** conidiomata produced on *Eucalytpus* stem sections on water agar **C, D** vertical section of conidioma **E** inner wall of conidioma **F** conidiomatal walls (ps, pseudoparenchymatous inner wall; pr, prosenchymatous outer or interlocular wall) **G** conidiogenous cells **H** conidia. Scale bars: 1 mm (**B**); 100 µm (**C, D**); 10 µm (**E–H**).

##### Sexual morph.

Not observed.

##### Asexual morph.

Produced after two months on *Eucalyptus* stem sections placed on water agar. *Conidiomata* superficial or with base embedded, singular or gregarious, pulvinate or conical with or without necks, often covered with mycelia, unilocular or multilocular, convoluted, 354–841 µm long, 185–654 µm wide. *Conidiomatal wall* outer or inter-locular stratum prosenchymatous; inner fertile layers pseudoparenchymatous, composed of several layers of flattened, thick-walled, pigmented cells, 8–19 µm thick. *Paraphyses* present, rarely observed. *Conidiophores* produced along inner layer of locule, simple or scarcely branched from basal cell, 8–11 µm long. *Conidiogenous cells* enteroblastic, lageniform, tapering towards apex, 5–11 × 1–2 µm. *Conidia* hyaline, oblong, straight or occasionally curved, 3–4 × 1–1.5 (3.2 ± 0.3 × 1.2 ± 0.07) µm.

##### Culture characteristics.

Colonies on 2% MEA, when young, showing circular growth with smooth edges, above white, reverse pale to dark brown (30 °C) and yellow (25 °C); with age, above becoming brown and reverse dark yellow; optimal growth at 30 °C (7.7 mm/d), followed by 25 °C (7.0 mm/d) and 20 °C (4.6 mm/d), minimal growth at 35 °C (0.1 mm/d), no growth at 5 °C; mycelia fluffy, density-sparse in centre, becoming thicker towards the edge, aerial hyphae more abundant at 25 °C than at 30 °C when young.

##### Habitat.

On/in bark of *Psidium cattleianum* and *Syzygium jambos*

##### Distribution.

Hawaii, USA

##### Notes.

*Celoporthe paradisiaca* is morphologically similar to its phylogenetically closest relatives, *Cel. hawaiiensis* and *Cel. guangdongensis*, but can be differentiated from them by DNA sequences. In the ITS, *BT1* and *TEF1* datasets, *Cel. paradisiaca* differs from *Cel. hawaiiensis* by 6, 3 and 3 base pairs and from *Cel. guangdongensis* by 7, 4 and 2 base pairs, respectively (Tables 6, 7).

### Pathogenicity tests

**Figure 6.**
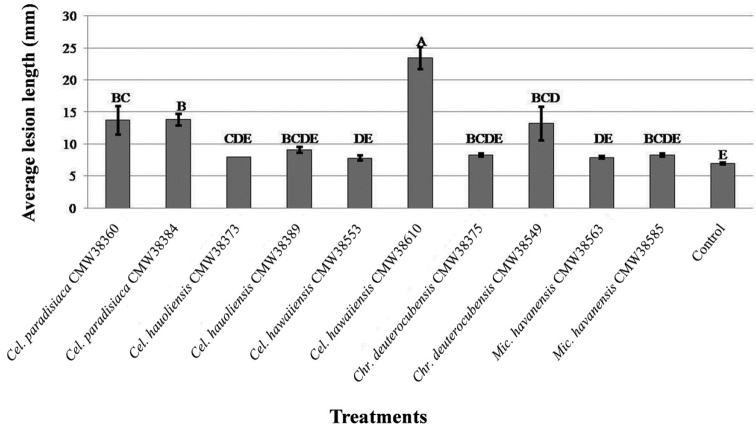
Vertical bar chart showing results of inoculation trial (xylem lesion) with Cryphonectriaceae isolates from Hawaii on *S. jambos* trees. Means with similar letters are not statistically significant, while those with different letters are statistically significant (significance level = 0.05).

Inoculation with two isolates each of *Chr. deuterocubensis* (CMW38375, CMW38549), *Mic. havanensis* (CMW38563, CMW38585), *Cel. hawaiiensis* (CMW38553, CMW38610), *Cel. hauoliensis* (CMW38373, CMW38389) and *Cel. paradisiaca* (CMW38360, CMW38384) resulted in lesions on the cambium of one-year-old *S. jambos* trees. There were no significant differences between the means for *Cel. hauoliensis* and *Mic. havanensis* when compared to the negative control (Fig. 6). There were significant differences in the means for *Chr. deuterocubensis* and *Cel. hawaiiensis* when compared with one another, as well as with the negative control. A strain (CMW38610) of *Cel. hawaiiensis* was the most pathogenic (Mean = 23.4 mm) of all the fungi tested and it resulted in a mean lesion length that was statistically different when compared to the means for other test strains and the negative control (Fig. 6). The inoculated fungi were re-isolated from the treated plants and not from the controls, thus fulfilling the requirements of Koch’s Postulates.

### Genetic Diversity of *Chr. deuterocubensis* isolates

*Chrysoporthe deuterocubensis* was the most commonly isolated fungus from Myrtales in this study (Table 1). Due to its known importance as a plantation tree pathogen, isolates obtained were subjected to a genetic diversity test using previously-developed microsatellite markers for this fungus. Seven of the 10 microsatellite primers amplified the desired target loci in 93 isolates obtained from four tree species on three Islands of Hawaii (Table 1). Allele sizes at each locus were estimated and these were within the size ranges for each marker (Van der Merwe et al. 2003). A total of seven alleles (one allele at each locus) and one haplotype were identified in the collection. The gene diversity was zero and the *Chr. deuterocubensis* collection from Hawaii was determined as 100% clonal.

## Discussion

Five species of Cryphonectriaceae, residing in the genera *Celoporthe*, *Chrysoporthe* and *Microthia*, were identified from native and non-native Myrtaceae from three of the Hawaiian Islands (USA). Of these, only *Chr. deuterocubensis* and *Mic. havanensis* have previously been found in Hawaii (Gryzenhout et al. 2006a, 2009; Van der Merwe et al. 2010). In addition, three new species of *Celoporthe* were discovered and described.

*Chrysoporthe deuterocubensis* is known to occur in Hawaii where it has been previously recorded as a pathogen of *Eucalyptus* trees from the Islands of Kauai and Hawaii (Hodges et al. 1979; Gryzenhout et al. 2009; M.J. Wingfield, unpubl.). This fungus, originally known as *Chr. cubensis* and later recognised as distinct from that species (Van der Merwe et al. 2010), is well-known from many south-eastern Asian countries where it is believed to have originated (Zhou et al. 2008; Gryzenhout et al. 2009; Chen et al. 2010; Van der Merwe et al. 2010; Wang et al. 2020). It exclusively infects trees in the Myrtaceae and is an important pathogen of *Eucalyptus* outside the native range of this tree (Gryzenhout et al. 2009; Van der Merwe et al. 2010).

The occurrence of *Chr. deuterocubensis* on native Ohia (*M. polymorpha*) in Hawaii could be of concern given its importance as a tree pathogen. This prompted us to investigate the population diversity of the fungus in Hawaii and, thus, to gain insights into its possible origin and movement in the region. The seven microsatellite markers, used to study the population diversity of *Chr. Deuterocubensis*, amplified target loci in ninety-three isolates of the fungus. The trees from which isolates were obtained represented three genera and four different species. The single isolate of the fungus from native *M. polymorpha* was also included. All isolates, irrespective of the host or island on which they were collected, represented a single genotype of *Chr. deuterocubensis* and further comparisons were not justified. Overall, the results of this study provide convincing evidence that *Chr. deuterocubensis* has been introduced into Hawaii.

The occurrence of a single clone of *Chr. deuterocubensis* in Hawaii is consistent with that of an introduced pathogen that would be expected to have low gene diversity. This is in contrast to native pathogens that are typically genetically diverse in their areas of origin (Gordon et al. 1996; McDonald 1997). The area of origin of *Chr. deuterocubensis* in Hawaii is unknown, but it is most likely some part of Asia where the pathogen is found on native, as well as non-native, Myrtales (Van der Merwe et al. 2010). The discovery of only a single genotype of *Chr. deuterocubensis* in Hawaii was surprising and unexpected. This is especially because the isolates were collected from a wide range of different trees spanning three genera and four species and occurring on three different Islands.

*Chrysoporthe deuterocubensis* has been known on *Eucalyptus* in Kauai (as *Cryphonectria cubensis*) for many years (Hodges et al. 1979; Gryzenhout et al. 2004) and this could be the area where it was first introduced. The pathogen also occurs on highly sought-after ornamental trees/shrubs, such as *Tibouchina* species (Myrtales: Melastomataceae) (Myburg et al. 2003; Gryzenhout et al. 2009) and it is believed to have been moved on cuttings of this tree (Myburg et al. 2003; Gryzenhout et al. 2009). *Tibouchina* is commonly grown in Hawaii and these trees could also represent a source of a first introduction. This would be in contrast to other Myrtales, such as *Eucalyptus* spp., that are more commonly moved as seed.

*Chrysoporthe deuterocubensis* is an aggressive and important pathogen of trees in the Myrtales. It is clearly widespread in Hawaii and it has most likely been present in the state for many years. It appears that the population of the pathogen has increased substantially where it infects *S. jambos*, apparently being pre-disposed to the development of the canker pathogen by rust caused by *A. psidii*. Once large populations of a pathogen, such as *Chr. Deuterocubensis*, develop in an area, the chance of their moving to new environments is heightened by what has been termed a “bridgehead effect” and for which there are numerous examples in *Eucalyptus* forestry (Wingfield et al. 2013, 2015).

*Microthia havanens*, found in this study on *P. cattleianum*, *S. jambos* and *S. cumini*, was first described as a saprobe on *Eucalyptus* trees and other trees such as Mango [*Mangifera indica* L. (Anacardiacae, Sapindales)], avocado [*Persea americana* Mill. (Lauraceae, Laurales)] and Jobo trees [*Spondias mombin* L. (Anacardiaceae, Sapindales)] in Cuba (Bruner 1916). Other hosts and areas of occurrence for this fungus include *Eucalyptus* in Mexico and Hawaii, *Myrica faya* Ait (Myricaceae, Fagales) trees in Madeira and the Azores (Gryzenhout et al. 2006a) and *Eucalyptus grandis* Hill: Maiden trees in Florida (USA) (Barnard et al. 1987). *Microthia havanensis* is considered a weakly pathogenic bark-infecting fungus. This was also confirmed in our pathogenicity studies on *S. jambos*, where the two isolates tested produced lesions that did not differ significantly from the controls.

Three new species of *Celoporthe* were found in this study, with thirteen species now recognised in the genus. These include ten species, *Cel. borbonica*, *Cel. cerciana*, *Cel. eucalypti*, *Cel. guangdongensis*, *Cel. hauoliensis*, *Cel. hawaiiensis*, *Cel. indonesiensis*, *Cel. paradisiaca*, *Cel. syzygii* and *Cel. tibouchinae* in the Asian clade (Chen et al. 2011; Ali et al. 2018; Wang et al. 2018) and three species, *Cel. dispersa*, *Cel. fontana* and *Cel. woodiana* in the African clade of this genus (Nakabonge et al. 2006; Vermeulen et al. 2013). The present study expands the species diversity and geographic range of *Celoporthe*.

Preliminary pathogenicity trials on *S. jambos* showed that some of the isolates of *Chrysoporthe* and *Celoporthe*, tested under greenhouse conditions, can result in significant lesions on inoculated plants within a short period of time. Both isolates of *Cel. paradisiaca* caused distinct lesions, while one isolate each of *Cel. hawaiiensis* and *Chr. deuterocubensis* resulted in lesions that were significantly larger than those of the controls. One of the *Cel. hawaiiensis* isolates was the most aggressive fungus tested and surprisingly more so than the well-recognised pathogen *Chr. deuterocubensis*. This fungus clearly deserves further study.

*Austropuccinia psidii* infects mostly young, actively growing leaves and shoots, as well as fruits and sepals (Coutinho et al. 1998; Alfenas et al. 2004; Glen et al. 2017). Infections of leaves and meristems are severe on susceptible seedlings, cuttings, young trees and coppice, causing plants to be stunted and multibranched, inhibiting normal growth and development and sometimes causing death to young seedlings (Booth et al. 2000; Rayachhetry et al. 2001). This is in contrast to species of the Cryphonectriaceae that infect the bark of trees and shrubs (Gryzenthout et al. 2009). *Chrysoporthe* species, for example, infect the bark and cambium of trees, giving rise to rapidly-expanding cankers on the stems (Gryzenhout et al. 2009). These cankers often girdle the stems, killing the cambium and leading to rapid tree death (Hodges et al. 1976; Wingfield et al. 1989; Gryzenhout et al. 2009).

In the surveys conducted in this study, samples with symptoms of the Cryphonectriaceae were obtained from various parts of trees, including dead branches, stem cankers and also on trees with no obvious infection by the myrtle rust pathogen, *A. psidii*. We believe that the rapid die-back of *S. jambos* trees and other non-native myrtles in Hawaii is, at least in part, due to infection by one or more Cryphonectriaceae species that apparently proliferate in tissue stressed by the Myrtle rust fungus.

## Supplementary Material

XML Treatment for Celoporthe hauoliensis

XML Treatment for Celoporthe hawaiiensis

XML Treatment for Celoporthe paradisiaca

## Supplementary materials

Supplementary material 1 Table S1 Data type PCR-based microsatellite markers Explanation note List of PCR-based microsatellite markers used in this study. https://binary.pensoft.net/file/492848This dataset is made available under the Open Database License (http://opendatacommons.org/licenses/odbl/1.0/). The Open Database License (ODbL) is a license agreement intended to allow users to freely share, modify, and use this Dataset while maintaining this same freedom for others, provided that the original source and author(s) are credited.Jolanda Roux, Gilbert Kamgan Nkuekam, Seonju Marincowitz, Nicolaas A. van der Merwe, Janice Uchida, Michael J. Wingfield, ShuaiFei Chen

Supplementary material 2 Table S2 Data type datasets and statistics Explanation note Datasets used and the statistics resulting from the phylogenetic analyses. https://binary.pensoft.net/file/492849This dataset is made available under the Open Database License (http://opendatacommons.org/licenses/odbl/1.0/). The Open Database License (ODbL) is a license agreement intended to allow users to freely share, modify, and use this Dataset while maintaining this same freedom for others, provided that the original source and author(s) are credited.Jolanda Roux, Gilbert Kamgan Nkuekam, Seonju Marincowitz, Nicolaas A. van der Merwe, Janice Uchida, Michael J. Wingfield, ShuaiFei Chen
