# Supplementary material for: Cryphonectriaceae associated with rust-infected Syzygium jambos in Hawaii
Source: MycoKeys. 2020 Dec 31;76:49–79. doi: 10.3897/mycokeys.76.58406 (PMC7790811; doi:10.3897/mycokeys.76.58406)
Supplement: Supplementary material 1 — Table S1 [file mycokeys-76-049-s001.docx]

Supplementary Table S1. List of PCR-based microsatellite markers used in this study.

| Locus | Dye | Primer | Primer sequence (5’-3’) | Bin size range |
| --- | --- | --- | --- | --- |
| CcPMC | VIC | PMCF | ttgcgtatggaaatgacg | 190–212 |
|  |  | PMCR | atggcgcttgtatagagca |  |
| CcPMG | 6-FAM | PMGF | tgattcacgtctattgccac | 197–297 |
|  |  | PMGR | gttaagttctcggtgaatcg |  |
| COL6 | 6-FAM | COL6F | ggccagggcagaggtaaggcag | 260–270 |
|  |  | COL6R | gctagagagtcaacatgatgtg |  |
| COL7 | VIC | COL7F | gaaccccgactacgtgattatc | 173–174 |
|  |  | COL7R | tggcactatatcaccatcactg |  |
| COL11 | VIC | COL11F | ctcatgggtccctgcatgcgac | 258–267 |
|  |  | COL11R | gtggcactaccagaacatacag |  |
| SA1 | NED | SA1F | ggaatcaccaccactagcgtcc | 300–320 |
|  |  | SA1R | gtgtctccgttaacgcagtggt |  |
| SA3 | 6-FAM | SA3F | tcaccaccactggcgtccagac | 200–215 |
|  |  | SA3R | tcgttatcttggtgactgtaga |  |
| SA4 | PET | SA4F | cagagcatgagatgaatagatg | 150–200 |
|  |  | SA4R | agtcaggctcttcacgctctgt |  |
| SA6 | PET | SA6F | atcgacgatcaggttctggatc | 209–221, 316–365 |
|  |  | SA6R | tattgcggtaacccaattttcg |  |
| SA9 | NED | SA9F | gctcgggctgccaatccttaag | 190–200, 203–215 |
|  |  | SA9R | cgccgagtttctcgccaccatc |  |
