# Supplementary material for: Cryphonectriaceae associated with rust-infected Syzygium jambos in Hawaii
Source: MycoKeys. 2020 Dec 31;76:49–79. doi: 10.3897/mycokeys.76.58406 (PMC7790811; doi:10.3897/mycokeys.76.58406)
Supplement: Supplementary material 2 — Table S2 [file mycokeys-76-049-s002.docx]

Supplementary Table 2. Datasets used and the statistics resulting from the phylogenetic analyses.

| Dataset | No. oftaxa | No. of bp^1^ | Maximum parsimony | | | | | | |
| --- | --- | --- | --- | --- | --- | --- | --- | --- | --- |
|  |  |  | PIC^2^ | No. of trees | Tree length | CI^3^ | RI^4^ | RC^5^ | HI^6^ |
| ITS+*BT1* | 117 | 1069 | 505 | 76 | 2239 | 0.442 | 0.886 | 0.392 | 0.558 |
| ITS | 29 | 512 | 97 | 552 | 138 | 0.841 | 0.944 | 0.794 | 0.159 |
| *BT1* | 29 | 416 | 56 | 15 | 79 | 0.873 | 0.940 | 0.821 | 0.127 |
| TEF | 25 | 280 | 75 | 6 | 95 | 0.895 | 0.953 | 0.853 | 0.105 |
| ITS+BT2+TEF | 29 | 1208 | 228 | 11 | 322 | 0.839 | 0.933 | 0.782 | 0.161 |

| Dataset | Maximum likelihood | | | | | | | | | | |
| --- | --- | --- | --- | --- | --- | --- | --- | --- | --- | --- | --- |
|  | Subst. model^7^ | NST^8^ | Rate matrix | | | | | Ti/Tv ratio^9^ | p-inv | Gamma | Rates |
| ITS+*BT1* | TPM3uf+I+G | 6 | 0.748 | 3.191 | 1.000 | 0.748 | 3.191 | – | 0.365 | 0.484 | gamma |
| ITS | TrNef+G | 6 | 1.000 | 1.930 | 1.000 | 1.000 | 3.417 | – | 0 | 0.267 | gamma |
| *BT1* | TrN+I | 6 | 1.000 | 2.024 | 1.000 | 1.000 | 5.567 | – | 0.772 | – | equal |
| TEF | TPM2uf | 6 | 2.828 | 6.412 | 2.828 | 1.000 | 6.412 | – | 0 | – | equal |
| ITS+*BT1*+TEF | TrN+G | 6 | 1.000 | 2.527 | 1.000 | 1.000 | 3.722 | – | 0 | 0.154 | gamma |

^1^bp = base pairs.

^2^PIC = number of parsimony informative characters.

^3^CI = consistency index.

^4^RI = retention index.

^5^RC = rescaled consistency index.

^6^HI = homoplasy index.

^7^model = best-fit substitution model.

^8^NST = number of substitution rate categories.

^9^Ti/Tv ratio = transition/transversion ratio.
